# Discovery of Knowledge in the Incidence of a Type of Lung Cancer for Patients through Data Mining Models

**DOI:** 10.1155/2022/6058213

**Published:** 2022-05-31

**Authors:** Yousif Saleh Ibrahim, Yasser Muhammed, Asaad T. Al-Douri, Muhammad Shahzad Faisal, Abdulsattar Abdullah H. Mohamad, Abdallah Al-Husban, Mequanint Birhan

**Affiliations:** ^1^Department of Medical Laboratory Techniques, Al-Maarif University College, Al-Anbar, Iraq; ^2^College of Technical Engineering, Al-Farahidi University, Baghdad, Iraq; ^3^Department of Dental Industry, College of Medical Technology, Al-Kitab University, Altun Kupri, Iraq; ^4^COMSATS University Islamabad, Attock Campus, Punjab, Pakistan; ^5^The University of Mashreq, Research Center, Baghdad, Iraq; ^6^Department of Medical Laboratory Techniques, Dijlah University College, Baghdad 10021, Iraq; ^7^Department of Mathematics, Faculty of Science and Technology, Irbid, Jordan; ^8^National University, P.O. Box: 2600, Irbid, Jordan; ^9^Department of Mechanical Engineering, Mizan-Tepi University, Tepi, Ethiopia

## Abstract

This paper presents the research results on the contribution of user-centered data mining based on the standard principles, focusing on the analysis of survival and mortality of lung cancer cases. Researchers used anonymized data from previously diagnosed instances in the health database to predict the condition of new patients who have not had their results yet. Medical professionals specializing in this field provided feedback on the usefulness of the new software, which was constructed using WEKA data mining tools and the Naive Bayes method. The results of this article provide elements of interest to discuss the value of identifying or discovering relationships in apparently “hidden” information to propose strategies to counteract health problems or prevent future complications and thus contribute to improving the quality of care. Life of the population, as would be the case of data mining in the health area, has shown applicability in the early detection and prevention of diseases for the analysis of genetic markers to determine the probability of a satisfactory response to medical treatment, and the most accurate model was Naive Bayes (91.1%). The Naive Bayes algorithm's closest competitor, bagging, came in second with 90.8%. The analysis found that the ZeroR algorithm had the lowest success rate at 80%.

## 1. Introduction

Defining the causes by which a disease is generated has become a task involving different areas of knowledge such as medicine, biology, applied mathematics, and computer science, generating new studies based on large amounts of medical information that help discover causes relevant to the behavior of the disease. Lung cancer is the most common cancer affecting both men and women [1]. The incidence has increased rapidly during the last four decades. It is more frequent in men. However, the number of cases in women continues to increase. Mortality tends to be higher in men, although it can vary according to the different geographical areas. Treatment for lung cancer is multidisciplinary and varies according to histological type, mutation profile, and clinical stage. It is necessary to have a multidisciplinary evaluation with various specialists due to the complexity of the cancer patient—prevention measures such as eliminating smoking and screening methods in the population at risk [1]. About 70% of lung cancer patients are diagnosed with advanced disease at the time of diagnosis. Most patients are not suitable for curative treatment. Molecular characterization has led to the definition of new subgroups such as lung cancer mutated for epidermal growth factor receptor, lung cancer rearranged for analytic lymphoma kinase and ROS1 kinase domain, and PDL1 expression, which need specific treatments and strategies [2].

Data mining is the processing of data [3] to find behavior patterns useful for decision making; it is closely related to statistics by using sampling and data visualization and purification techniques and having databases as raw material. The data analysis can jointly provide true knowledge that helps in decision-making. In the study in 2015 [4], classification approaches were proposed to determine the degree of malignancy of small-sized lung nodules. The Lung Image Database Consortium (LIDC) database offered by the American Cancer Institute was used in the study. The LIDC database [5, 6] is a database that has been evaluated by radiologists from four different institutes and includes radiographic descriptive information as well as the degree of nodule malignancy. In the statistical significance tests, it was observed that the Random Forest- (RF-) based team classifier, which had the highest classification performance, was superior to other methods [7–9]. In 2018, an automatic nodule region detection method based on artificial intelligence and image processing techniques was developed using lung tomography. In this context, a method and system that provide automatic detection and diagnosis of nodule regions on lung CT cross section images have been developed [10, 11].

In the clinical scope, data mining results aid in identifying and diagnosing pathologies to discover possible correspondences between various diseases. The patient with lung cancer presents alterations in other health issues that must be considered; the management of pain and other symptoms represents only part of the help that can be given to improve the patient's quality of life. In this study, firstly, the data set of lung cancer disease was obtained by taking the physicians' opinions, and then models were created with this data set. As a result, the most successful algorithm among the models created was determined.

## 2. Methodology

In the study, first of all, the opinions of doctors working in the field of lung cancer were taken, and preliminary research was carried out for the data set suitable for this field. Data mining steps were applied to the appropriate data set. At this stage, preprocessing and data cleaning, data reduction, data transformation, and data mining operations were performed, respectively, and after these processes, an evaluation and conclusion were drawn, and the study was completed.

(1) Stage-1: the study was started by taking the doctors' opinions working on lung cancer and researching the data set suitable for this field. After the appropriate data set was found, data mining steps were applied before processing this data set. At this stage, data cleaning, data reduction, data conversion, and appropriate data mining software selection processes were carried out, respectively. Afterward, the feature analysis was made, the model creation part was started, and the most successful one among the compared models was selected and used in the software that produced the prediction result for diagnosis.
(2) The first step in the data mining process is data collection. The personal information of some patient candidates who applied to hospitals with the suspicion of Malignant Neoplasm of Bronchus and Lung (C34) cancer, which is in the health database in Baghdad, Iraq, was obtained anonymously and provided that their personal information is kept confidential. Many tests are used to diagnose lung cancer, but in this study, it was tried to draw a conclusion based on these parameters by using the parameters also referred to as the hierogram test.
(3) The next stage, the data cleaning stage, is extremely important for the success of data mining, and the success of this stage directly affects the success of the result. After the data collection phase, incompatible data, null values, and extreme values were removed from the data obtained. While the number of patient records drawn from the database is 700, the number used in the project is 404. Data with missing or outlier data were excluded from this analysis during the data cleaning phase to make the result more successful. After the cleaning process, the project was started with 404 patient data, of which 81 were C34, and 323 were not C34 (Control).
(4) In the data reduction phase, due to the presence of unnecessary data such as both birth date data and age data in the data set, the data that will adversely affect the working time of the computer and reduce the quality of the results, which express the same result, have been removed from the data set.
(5) In the data conversion phase, inconsistent data types encountered were corrected and incorrectly entered extreme values normalized for the data set to gain a standard structure. The data set, which was later transferred to excel format, was converted to *csv* format for easy data mining algorithms. In addition, at this stage, string-type data has been converted into numeric data. After these corrections were made in the data set, studies were carried out to select the appropriate data mining algorithm and the model creation phase. Various software tools are available for making data mining applications. The most commonly used ones in this field are *R programming*, *Rapid Miner*, and *WEKA*.
  - R Programming (RR): also known as R&R. R provides superiority to S language with different applications. It has linear and nonlinear modeling, classical statistical tests, time-series analysis, classification, and clustering algorithms. It can run on many operating systems.
  - RapidMiner (RM): RapidMiner also has a user-friendly interface and supports all types of files.
  - WEKA: WEKA is the most widely used data mining program today; it is an open-code program developed on the java platform. It is preferred because of its compatibility with all operating systems. WEKA includes data processing, classification, clustering, and data association features. This study preferred WEKA is a data mining tool since the WEKA integration with Java works better.

## 3. Application and Results

In this section, information about the lung cancer data set used, the features to be used in the study, the data mining algorithms used in the model creation phase, the software developed for this purpose, and the output of this software are explained.

### 3.1. Summary of Lung Cancer (C34) Dataset Used

Data mining is extracting and interpreting data from a digital environment. The data source must be created correctly and thoroughly [12]. The data of patients and patient candidates who sought treatment for lung cancer in Baghdad, Iraq, were acquired anonymously from the lung cancer database and used for data mining. Except for the governorate, municipality, gender, and age used in the study, the following attributes directly impact the findings. No matter how regular and dependable the data set is, it must be preprocessed before use. The data set was preprocessed and made suitable for model construction to improve the study's accuracy and success rate. Patient records with too many missing values were purged prior to the study to obtain a relevant result. After deleting the patient data, a new data set with 404 patient data was obtained. This component of the study includes information concerning nine patient features.

#### 3.1.1. Clinical Data of the Selected Patients

**Figure 1:**
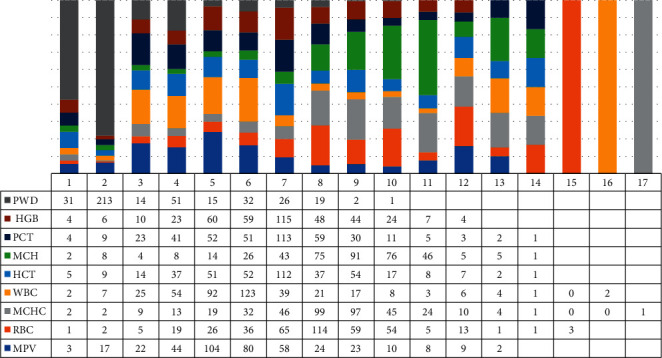
Clinical data of the selected patients.

Within the scope of the study, the diagram showing the distribution of various parameters along with their reference values in Figure 1, Platelet Distribution Width (PDW): 9–14, Red Blood Cells (RBC): 3.2–6, White Blood Cells (WBC): 3–12, Mean Corpuscular Hemoglobin Concentration (MCHC): 30–36, Mean Platelet Volume (MPV): 0–15, Hematocrit (HCT): 30–55, Hemoglobin (HGB): 10–18, Mean Corpuscular Hemoglobin (MCH): 25–33, and Procalcitonin (PCT): 0.19–0.36 values of 404 data in the data set is as seen in Figure 1.

Various software tools are available for making data mining applications. The most commonly used ones in this field are R programming, RapidMiner, and WEKA. They have advantages and disadvantages compared to each other. It can be said that each of the data mining software tools mentioned above has its own advantages. However, some were not preferred in this study because of the following: Yale is not easily accessible, R is not widely used on UNIX machines, and it is used with the help of experts on the windows system and does not have enough algorithms. Since the WEKA data mining tool is Java-based, WEKA was preferred as data mining software in this study, thinking that its integration would be more efficient.

#### 3.1.2. Attribute Analysis

After completing the basic stages such as data transformation and data cleaning, the feature analysis stage was started. Since the WEKA data mining program was preferred here, the data was converted into a format suitable for WEKA. The format suitable for WEKA is CSV and ARFF format. In this context, first of all, the cleaned data is given in Figure 1. The data saved as *∗* xls in Excel format was opened with a notepad and converted to CSV format. *∗* xls file is used to define the data saved with the CSV extension as WEKA's input data. “,” replaces the character, and “.” replaces the “,” character between numbers. The conversion is provided by typing the character. The data obtained after this stage were made suitable for working with WEKA called edited data set.

### 3.2. Generating Appropriate Model with Data Mining Algorithms

Various classified algorithms, frequently mentioned in the literature, which will be explained in detail in the following sections, were applied to the data set. The data set is divided into training and test sets to test the success of the method applied in data mining studies. This separation can be done in various ways. For example, one of the methods that can be used is to reserve 66% of the data set for training and 33% for testing and test the success of the test set after the training set, and the system is trained. Random assignment of these training and test sets is another method. However, in this study, 10 is the most preferred *k* value in the literature. The meanings of some of the lines expressing the accuracy and reliability stated here are given as follows:

 
*Correctly classified instances*: number and percentage of correctly classified instances.
 
*Mean absolute error*: average absolute error.
 
*True Positive (TP) Rate*: accurate prediction rate.
 
*False Positive (FP) Rate*: incorrect prediction rate.
 
*Confusion matrix*: it is the relationship matrix between predictions and actual values.
 
*Obtaining and evaluating model results*: algorithms frequently used in this field in the literature were applied to the data set. Screenshots and evaluation results of these will be explained in detail in the following sections. In this context, the success results for each algorithm will be compared.
 
*Choosing the most suitable model*: while choosing the appropriate model, algorithms frequently mentioned in the literature are included. The most successful algorithm was decided based on the correct prediction rate in this context. Models were created one by one with the selected algorithms, and as a result, it was decided to apply the Naive Bayes algorithm, which has the highest accuracy with 91.09%, to the data set. While choosing this algorithm, accuracy value, time, and average absolute error were taken into account.

### 3.3. Algorithm Design and Software Development Suitable for the Selected Model

As a result of using the algorithms in the WEKA data mining software on the data set, first of all, detailed information was obtained about the algorithm that can make the most appropriate prediction estimation. Afterward, this algorithm was accessed from the developed software, the model was created, and a meaningful result was tried to be obtained [13]. During the development of the software, attention was paid to ensuring that it had the following criteria:

- User-friendliness of the software
- User (doctor or health personnel) can enter blood value test results into the system simply.
- I am displaying some model values used, such as F-Measure, Precision, and Recall, on the interface for informational purposes.
- The user was asked to enter nine attributes to produce a result that can be understood and easily interpreted by the user according to the entered values with the software made in this context. As a result of these entered values, an estimate of the C34 disease diagnosis of the patient candidate is obtained. This is an informative result for doctors and healthcare professionals.

#### 3.3.1. Creating an Interface with the Developed Software and Operating the System

This study aims to produce the most accurate prediction by using the data mining method for the diagnosis of lung cancer. With the most successful one among the examined algorithms, a study has been made to create an interface that the health personnel will use that can produce meaningful predictions for the personnel by taking nine input values that will enable this algorithm to work. After studies and research were made for the interface to be created, it was decided to develop the interface with Java. From this interface screen, nine attribute values will be entered, and then the system will produce a prediction result for the diagnosis of lung cancer with the evaluate button. In the developed tool, a user-friendly interface has been designed so that the test can be applied easily and easily understood by each doctor or healthcare worker. After the data requested by the healthcare professionals are entered into the system, the developed algorithm will run, and the prediction about the disease will be presented to the healthcare personnel in the line indicated as C34 Status. This information is of great importance in giving doctors and healthcare professionals an idea.

#### 3.3.2. Models and Performance Measures Created on Lung Cancer Dataset with WEKA

Influencing the analysis result, such as the features selected in the data preprocessing process, the completion of missing data, the correction of extreme values, and the removal of many rows from the data set due to the large number of data being NULL directly affect the model extraction. A different preprocessing process will also affect the success of the model [14].

In this study, many models were created with different algorithms to predict whether a person has lung cancer with the data set consisting of records that have and have not been diagnosed with lung cancer. In this section, the performance levels of the models created are compared. J48 and RandomTree, which are decision tree algorithms in practice and based on ID3 and C4.5 algorithms, are among the Bayesian Classification algorithms, a statistical algorithm. naïve Bayes and BayesNet, sample-based classification algorithms Kstar, regression-based algorithms, Logistic Regression, and Multilayer Perceptron models were created using Bagging, One R, and Zero R algorithms, and the performance grades of these models were compared.

#### 3.3.3. Naive Bayes Algorithm and Performance Criterion [15]

**Figure 2:**
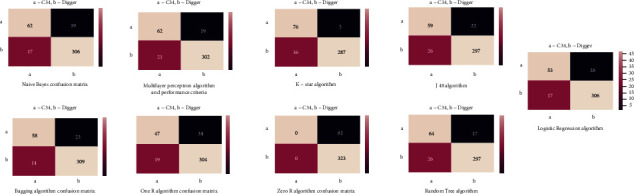
Confusion matrix for selected algorithms.

**Figure 3:**
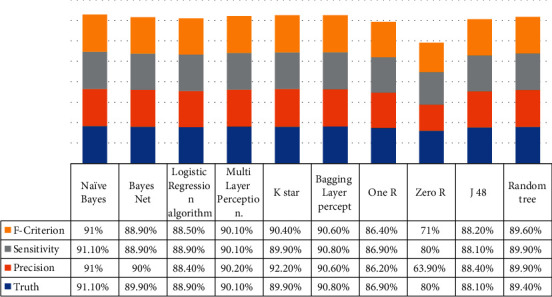
Comparison criteria of the various created models.

After the data set was converted to CSV format that the WEKA program could read, model creation was carried out. The statistics and confusion matrix of the test results of the model created with the Naïve Bayes algorithm, which is a statistical algorithm that classifies datasets and classifies unknown data on the basis of probability, is shown in Figure 2, and the comparison criteria of the algorithm's model are shown in Figure 3. In Table 1, 62 of 81 pieces of C34 data were classified correctly, and 306 of 323 pieces of other data were classified correctly, resulting in an accuracy rate of 91.1% as shown in Figure 3.

#### 3.3.4. Logistic Regression Algorithm and Performance Measure

Another algorithm applied for comparison purposes is the logistic regression algorithm, which is one of the regression-based methods. After applying this algorithm to the data set, Figure 3 and Table 1 were obtained. 53 of 81 pieces of C34 data were classified correctly, and 306 of 323 pieces of other data were classified correctly, resulting in an accuracy rate of 88.9% as shown in Table 1.

*(1) Comparison of Created Models*. Naive Bayes, BayesNet, Logistic Regression, Multilayer Perceptron, KStar, Bagging, OneR, ZeroR, J48, and finally Random Tree algorithms were applied to the preprocessed data source, and models were created. Statistical information, confusion matrices, and comparison criteria of these models are shown in the previous section in detail in Figure 3. To make a better comparison, the accuracy, precision, sensitivity, and *F*-Measure values of each model are shown in Figure 3. When the values in Figure 3 are examined, it can be said that the Naive Bayes algorithm produces the best result with an accuracy rate of 91.1%. The accuracy criterion is the most basic and, at the same time, the most important criterion. According to this criterion, the Naive Bayes algorithm is followed by Bagging, Multilayer Perceptron, K star and Bayes Net, Random Tree, Logistic Regression, J48, One R, and Zero R algorithms, respectively.

#### 3.3.5. Multilayer Perceptron Algorithm and Performance Criteria

Multilayer Perceptron consists of the input layer with the input neurons, the output layer with the output neurons, and one or more hidden layers. The input layer receives the inputs from the multilayer network and transmits them to the middle layer, the processing elements in this layer are connected to all the processing elements in the next intermediate layer, and the algorithm works in this way. The Multilayer Perceptron algorithm, generally used to solve nonlinear problems, was applied to the data set used in the study, and a model was created. The confusion matrix of this model is shown in Figure 2, and the comparison criteria are shown in Figure 3. In Table 1, 62 out of 81 pieces of C34 data were classified correctly, and 302 out of 323 pieces of other data were classified correctly, resulting in an accuracy rate of 90.1% as shown in Table 1. K Star algorithm and performance criteria: in this section, the model creation process was carried out with the KStar algorithm, which is an example-based learning algorithm, which is widely used in the literature and is also included in WEKA. The confusion matrix of this model is given in Figure 2, and the information including the comparison criteria is given in Figure 3. In Table 1, 76 out of 81 pieces of C34 data were classified correctly, and 287 out of 323.

#### 3.3.6. Bagging Algorithm and Performance Criterion

Another data mining algorithm applied to the data set is the Bagging algorithm, which is among the subheadings of the Meta tab in WEKA. This algorithm was first proposed by [7–9, 16–20]. In Bagging, a training set with *N* samples is produced from the training set consisting of *N* samples, by random selection with substitution. In this case, some training samples are not included in the new training set (approximately 33%), while some are included more than once. Each basic learner in the community is trained with training sets containing different examples produced in this way, and the results are combined with majority voting. The confusion matrix of the model created with the Bagging algorithm is presented in Figure 2, and the comparison criteria are presented in Figure 3. In Table 1, C34 data were classified correctly, and 309 of 323.

#### 3.3.7. OneR Algorithm and Performance Criterion

The OneR algorithm (single class limitation) is one of the algorithms found under the subheadings of the rules tab in WEKA, which creates rule trees by testing a feature and is an algorithm that is frequently mentioned in the literature. For this reason, it was preferred to create models and compare them in the solution phase of the problem. The confusion matrix of the model created with this algorithm is given in Figure 2, and the comparison criteria are given in Figure 3. In Table 1, 47 of 81 pieces of C34 data were classified correctly, and 304 of 323 pieces of other data were classified correctly, resulting in an accuracy rate of 86.9% as shown in Table 1.

#### 3.3.8. ZeroR Algorithm and Performance Criterion

ZeroR algorithm is a simple algorithm. This algorithm estimates the average value of numerical or nominal test data. It applies the rules of basic coverage algorithms [21]. ZeroR only tries to detect the majority class distribution, which is assumed as a rule. In ZeroR, the arithmetic mean for numeric class attributes and the mod class value for nominal class attributes are estimated. ZeroR does not produce any rules other than this [22]. The confusion matrix of the model created with this algorithm is given in Figure 2, and the comparison criteria are given in Figure 2. In Table 1, all of the 81 C34 data were misclassified, and all of the 323 other data were classified correctly.

#### 3.3.9. J 48 Algorithm and Performance Criterion

In this section, a model was created on the data set with the J48 algorithm, which is a decision tree algorithm and is based on ID3 and C4.5 algorithms. The confusion matrix of this created model is shown in Figure 2, and the comparison criteria are shown in Figure 3. In Table 1, 59 out of 81 pieces of C34 data were classified correctly, and 297 out of 323 pieces of other data were classified correctly, resulting in an accuracy rate of 88.1%, as shown in Table 1.

#### 3.3.10. Random Tree Algorithm and Performance Criteria

Random Tree algorithm, another decision tree algorithm frequently used in the literature, was used for comparison purposes during model creation. The confusion matrix of the model created with this algorithm is shown in Figure 2, and the comparison criteria are shown in Figure 3. In Table 1, 64 out of 81 pieces of C34 data were classified correctly, and 297 out of 323 pieces of other data were classified correctly.

## 4. Discussion and Findings

Data mining aims to extract meaningful information from large data piles in the digital environment and evaluate this information. The biggest problem in studies conducted for this purpose is that data sources contain incorrect data in large data stacks, or the values of many attributes are entered incompletely. These problems were encountered in the data set used in this study. In terms of the reliability of the application results, the data set was subjected to a detailed preprocessing process. At this stage, in the data set, many processes such as cleaning the data, removing the inconsistent and noisy data from the data set, deleting the rows with too many null values from the data set, pruning the extreme values, and data reduction were applied.

In data mining, different methods are used in the stage of accessing information. There are many algorithms for these methods. There are many studies on which of these algorithms are more successful, and the results of these studies differ from each other. The main reason for this is that this success depends on the data set used, the preprocessing process of the data, and the selection of algorithm parameters. It is normal for different researchers to have different results of studies with different parameters on different data sets. In this study, Naive Bayes, BayesNet, Logistic Regression, Multilayer Perceptron, KStar, Bagging, OneR, ZeroR, J48, and Random Tree algorithms were applied to the data set, and a model was created with each algorithm. The most successful result was obtained with the Naive Bayes algorithm among these models. This algorithm was used in the software developed because of the success rate of 91.1% with Naive Bayes. The developed software is 91.1% successful in giving ideas to physicians.

### 4.1. Survey Study on Physician's Opinions

A questionnaire with evaluation criteria between 1 and 5 was sent to professionals in the field to get their feedback on the system. Using data from several hospitals, 20 physicians were asked for their thoughts, and the average score for each question was calculated. In this context, the survey questions and the results of the physicians' responses to the survey questions were evaluated. So, 4 and 5 points are green, 1 and 2 are red, and 3 are yellow. However, question 3, which asks how many elements the study will have, has a lower score than the other questions, as shown in the table. Some doctors claimed that more qualities would be more useful in treating the situation at this time. On the other hand, the third question had the lowest average score of 3.4, indicating that some doctors felt that the number of qualities should be increased, while others felt that it was adequate.

## 5. Conclusion and Recommendations

This study used a suggestion system to help diagnose lung cancer. Various algorithms were applied to the data set to construct models. Java software was developed using the most successful algorithm, and the software presented to healthcare professionals was obtained. After the data was entered by the data entry employees, they were examined one by one and turned into a single standard, and models were constructed on the data set and compared. Models developed with several algorithms were compared to see which algorithm produced the best model. The model was built using WEKA data mining. Ten algorithms were chosen from the WEKA data mining algorithms for comparison based on their popularity and literature studies. A Random Tree algorithm combines Naive Bayes, BayesNet, Multilayer Perceptron, KStar, Bagging, OneR, ZeroR, and J48. The Naive Bayes algorithm, which is a statistical algorithm, provides a better model than other algorithms when these models are compared. The most accurate model was Naive Bayes (91.1%). The Naive Bayes algorithm's closest competitor, Bagging, came in second with 90.8%. The analysis found that the ZeroR algorithm had the lowest success rate at 80%.

Using the provided interface, the software creates an estimate for the C34 diagnosis for the user based on the values entered. It has an adaptable, updateable, user-friendly interface and accessible structure to keep up with technological developments. Using the Naive Bayes method, this study had a 91.1% success rate compared to other studies in this sector.

### 5.1. Recommendations

This study can be done on datasets in different categories, it can be used to identify different cancer types, they can be compared using more algorithms, the study can be expanded by using a different data mining tool other than WEKA, or the number of features and data used in the study can be increased to be obtained in a different study in this area, and the accuracy can be evaluated.

## Figures and Tables

**Table 1:** Comparative analysis of other information about the various created models.

	Logistic regression algorithm	Naive Bayes algorithm	K star	Bagging algorithm	OneR algorithm	ZeroR algorithm	J48 algorithm	Random tree	. Naive Bayes
Correctly classified instances	88.86%	89.86%	88.87%	90.84%	86.87%	79.90%	88.20%	89.01%	89.01%
Kappa statistics	0.64	0.69	0.63	0.7	0.6	0	0.64	0.68	0.68
Mean absolute error	0.16	0.11	0.156	0.153	0.14	0.31	0.13	0.1	0.1
Root mean squared error	0.3	0.3	0.288	0.28	0.4	0.401	0.34	0.33	0.33
Relative absolute error	49.30%	33.70%	49.30%	47.68%	40.79%	100.00%	38.40%	33.10%	33.10%
Root relative squares error	72.03%	70.00%	72.03%	67.54%	90.46%	100.00%	84.00%	82.00%	82.00%
Total number of Instances in all algorithms: 404									

## Data Availability

The data underlying the results presented in the study are available within the manuscript.
